# Evidence for a novel gene associated with human influenza A viruses

**DOI:** 10.1186/1743-422X-6-198

**Published:** 2009-11-16

**Authors:** Monica Clifford, James Twigg, Chris Upton

**Affiliations:** 1 Department of Biochemistry and Microbiology, University of Victoria, Victoria, BC, V8W 3P6, Canada

## Abstract

**Background:**

Influenza A virus genomes are comprised of 8 negative strand single-stranded RNA segments and are thought to encode 11 proteins, which are all translated from mRNAs complementary to the genomic strands. Although human, swine and avian influenza A viruses are very similar, cross-species infections are usually limited. However, antigenic differences are considerable and when viruses become established in a different host or if novel viruses are created by re-assortment devastating pandemics may arise.

**Results:**

Examination of influenza A virus genomes from the early 20^th ^Century revealed the association of a 167 codon ORF encoded by the genomic strand of segment 8 with human isolates. Close to the timing of the 1948 *pseudopandemic*, a mutation occurred that resulted in the extension of this ORF to 216 codons. Since 1948, this ORF has been almost totally maintained in human influenza A viruses suggesting a selectable biological function. The discovery of cytotoxic T cells responding to an epitope encoded by this ORF suggests that it is translated into protein. Evidence of several other *non-traditionally *translated polypeptides in influenza A virus support the translation of this genomic strand ORF. The gene product is predicted to have a signal sequence and two transmembrane domains.

**Conclusion:**

We hypothesize that the genomic strand of segment 8 of encodes a novel influenza A virus protein. The persistence and conservation of this genomic strand ORF for almost a century in human influenza A viruses provides strong evidence that it is translated into a polypeptide that enhances viral fitness in the human host. This has important consequences for the interpretation of experiments that utilize mutations in the NS1 and NEP genes of segment 8 and also for the consideration of events that may alter the spread and/or pathogenesis of swine and avian influenza A viruses in the human population.

## Background

Influenza A viruses have had, and continue to have, an extremely significant deleterious impact on human health [1,2]. In spite of huge research efforts, the development/deployment of vaccines and more recently anti-viral drugs [3-6], the regular occurrence of global pandemics and yearly epidemics generate levels of morbidity and mortality that unfortunately keep this virus among the "top" human pathogens[7]. However, this research effort has greatly expanded our understanding of influenza transmission [8-10], evolution [11] and pathogenesis [12-14].

Over the years, a large and valuable collection of influenza A virus genomic sequences has been acquired at NCBI [15] and BioHealthBase [16]. It has been mined extensively to correlate pathogenicity with RNA and encoded protein sequences, revealing much about the processes of antigenic shift and drift, the effect of which is that currently circulating influenza A virus may escape, to a greater or lesser degree, the protective effect of our immune system primed against a previous influenza A infection or vaccination. More recently, the application of new technologies to the problem has lead to determination of the genomic sequence of the infamous 1918 influenza A strain [17-19] and its subsequent reconstruction into a viable virus. However, the precise origin of the 1918 pandemic virus is still not clear, nor why it was so virulent.

Our current understanding of the influenza A virus is that it has a segmented (8 pieces) negative sense single-stranded RNA genome, which encodes 11 proteins[20]. Each genome segment is transcribed to produce a single capped mRNA species, which in the case of segments 7 and 8 also undergoes splicing so that each encodes 2 proteins, M1/M2 and NS1/NEP respectively[21]. The segment encoding PB1, a polymerase subunit, also generates an additional protein, PB1-F2, that is not translated from the first AUG of the mRNA, rather the PB1-F2 peptide is produced as a result of translation initiating at an alternate start codon in a different reading-frame to that used for PB1 [22]. The PB1-F2 peptide is present in most, but not all, influenza A virus isolates [23] and is an important virulence factor [22,24-26]; presumably it has evolved secondarily to the PB1 polymerase gene. However, influenza virulence is not tied to one or a few genes, there are multiple lines of evidence that most if not all of the influenza A proteins contribute to the pathogenicity of the virus in humans [18,27,28].

In this paper we provide multiple lines of evidence to support the hypothesis that a large Open Reading Frame (ORF), present on the negative, genomic, strand of influenza A virus segment 8 encodes a protein that provides a selective advantage to viruses that infect humans. As a result of the evolutionary selective process, almost every human influenza A virus isolated in the last 50 years possesses this ORF, excluding those that have recently been acquired from avian or swine hosts. Although we are not the first to observe this ORF, it has been rarely been commented upon by others. It was observed when segment 8 was first sequenced [29] and more recently, the ubiquity of this ORF was briefly noted after we began this work [30].

## Results

### Distribution of a large genomic strand ORF among influenza A viruses

It should be noted that the characterization of influenza A virus genomes was complicated by a variety of errors in the available data sets. There are a number of minor and major (those that break essential genes) sequencing errors including contamination of virus materials that lead to a number of mis-identified sequences [31]. Also, the convention of naming these viruses for the organism from which they were isolated complicates their classification; for example, many avian-derived H5N1 viruses are labeled as "human". By necessity, we have therefore excluded a small number of sequences that clearly contain sequencing errors from this analysis.

**Figure 1:**
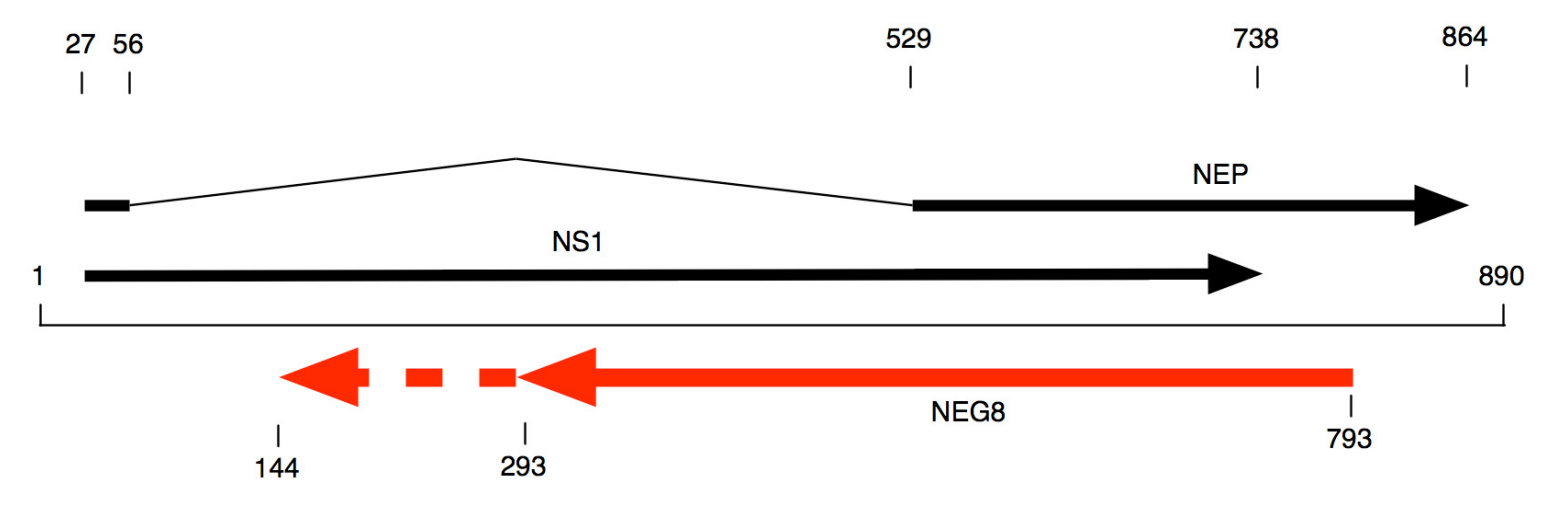
**Organization of the NS1 and NEP genes on genome segment 8**. The genome segment is from the 1918 human influenza A virus H1N1. The 167 codon NEG8 ORF is shown as a solid red arrow; the 168-216 region of the 216 codon NEG8 ORF is shown as a dashed red arrow.

While reviewing the genome sequence of the 1918 strain of influenza A virus (Accession no. AF333238; A/Brevig_Mission/1/18(H1N1)) for teaching purposes, one of us (CU) noticed the presence of an ORF capable of encoding 167 aa on the genomic (negative) strand of segment 8 (Figure 1). This struck us as being unusually large and lead us to wonder if it might be encoding a polypeptide even though no process for 1) translation of genomic RNAs or 2) generation of mRNAs with the same sequence as genome strands has been proposed for influenza A virus. A thorough survey and analysis of influenza A virus genomic sequences, together with a literature review yielded unexpected, but very interesting results.

**Figure 2:**
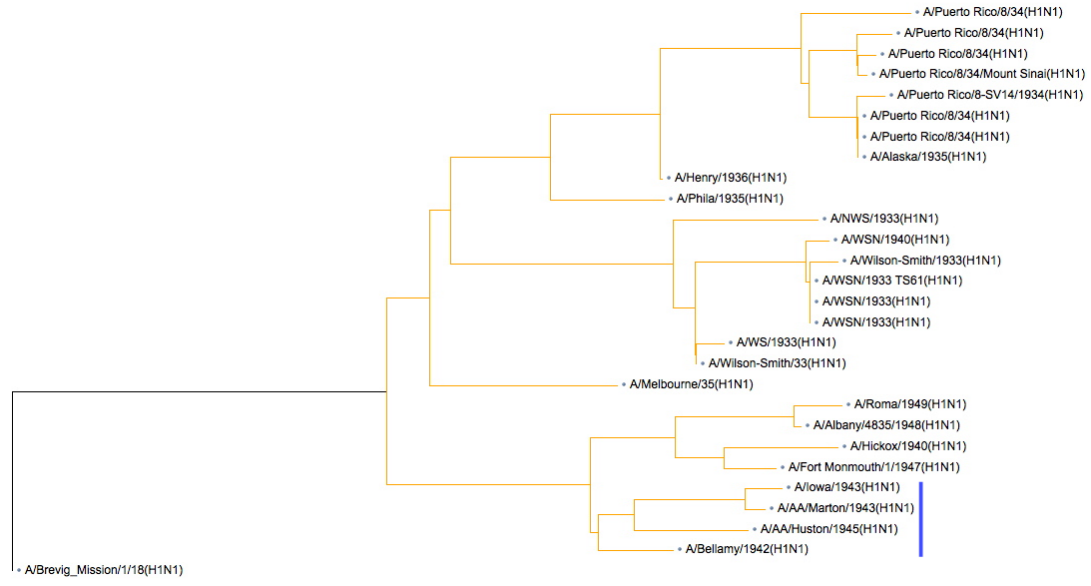
**Neighbour-joining tree constructed from pre-1950 human influenza A virus NS1 proteins using software at NCBI**. Blue bar indicates a single clade of viruses, which has no further descendents, that lack the 167 codon NEG8 ORF.

Influenza A viruses from the first half of the 20^th ^century were surveyed first; these sequences revealed that almost all the human viruses possessed an ORF on the negative strand of segment 8, which we call NEG8, that was at least 167 codons long. The 4 human viruses without this 167 codon ORF, due to mutation of the start codon, form a distinct clade (Figure 2); 1 member is A/bellamy/1942H1N1 (Accession no. M12596). These 4 viruses, which were isolated between 1942 and 1945, clearly demonstrate that this ORF is not absolutely essential for human influenza A viruses to 1) replicate and cause disease in humans, and 2) persist in the human population/environment and cause infection over several influenza seasons. However, the conservation of this 167 codon NEG8 ORF over almost 50 years suggested to us that it had a selectable role in the viral life cycle that maintained it in the human influenza A virus population. The data from this relatively small group of 53 virus isolates, including human, avian and swine viruses, also shows that similar segments, with 167 codon NEG8 ORFs were present in a small number of swine and avian influenza A viruses circulating in the same time period. Interestingly, the 1902 avian influenza A virus segment 8 sequences also possess the 167 codon NEG8 ORF, and therefore does not conflict with the hypothesis that one or more segments of the 1918 human influenza A virus were derived from avian influenza A virus source [17,18,32]. Similarly long ORFs were not observed for the other segments of the influenza A virus genome.

**Figure 3:**
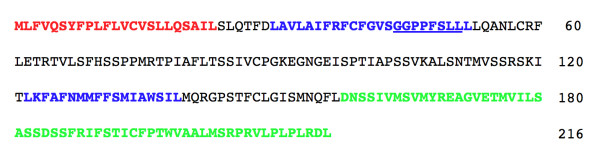
**Predicted protein sequence for a human influenza A virus 216 codon NEG8 ORF**. Consensus predictions, from multiple tools, for signal sequence and transmembrane domains are shown by colored letters. Red characters indicate the predicted signal sequence; Blue characters indicate the predicted transmembrane domains; Green characters indicate the polypeptide extension from 168 to 216 aa; the underlined characters indicate the functional CTL epitope.

This first analysis also revealed another subset of a 5 human influenza A viruses that possesses a mutation that changes the TAG stop codon of the NEG8 ORF into TAT, which is translated as tyrosine. These viruses form a distinct clade, with the earliest isolate, A/Albany/4835/1948H1N1 (Accession no. CY019951), collected in 1948. This mutation results in the extension of the NEG8 ORF to 216 codons (Figure 1). Since viruses that lost the 167 codon NEG8 ORF have not persisted in the human population more than a few years, it was of interest to examine the persistence of the mutation that extended the NEG8 ORF. A review of all viruses isolated after 1947 revealed that essentialy *all *subsequent human influenza A viruses have probably evolved from this group of viruses, or close relatives, which possess the 216 codon NEG8 ORF (Figure 3). Of 1739 true human influenza A viruses (all H and N types), meaning viruses isolated from humans but derived from birds (H5N1) or swine (current H1N1 pandemic virus) were excluded, isolated after 1950, all but 62 possess a 216 codon (or longer) NEG8 ORF. A description of the non-216 codon NEG8 ORFs, together with the number of each type is given in Table 1; these still possess the change that caused the original loss of the stop codon after the 167 codon ORF. An examination of this group of viruses shows that the variants arose from far fewer than 62 separate events and that within this group (non-216 codon ORF NEG8) there are actually only 2 examples that show sufficient persistence and spread in the human population, albeit very briefly, to be subsequently re-isolated from other individuals with influenza.

**Table 1:** Analysis of human influenza virus NEG8 ORFs, post 1950, which vary from the 216 codon length

Length of NEG8 ORF (codons)	No. of viruses	Description of ORF
261	2 (.12%)	Loss of stop codon from 216 codon ORF; ORF runs to end of sequence

258	1 (.06%)	Loss of stop codon from 216 Codon ORF; ORF runs to end of sequence

246	4 (.23%)	Loss of stop codon from 216 Codon ORF.

235	1 (.06%)	Loss of stop codon before 216 ORF start codon; extends 5' end of ORF.

204	1 (.06%)	Mutation resulting in new stop codon.

197	22 (1.3%)	Mutation resulting in new stop codon; not 22 separate events.^1^

178	1 (.06%)	Mutation resulting in new stop codon.

167	2 (.12%)	Most closely related to viruses isolated in 1930s; contamination resulting in mis-named viruses.

147	2 (.12%)	Mutation resulting in new stop codon; 2 separate events.

142	20 (1.1%)	Loss of start codon or new stop codon close to start of NEG 8 ORF.^2^

140	2 (.12%)	Mutation resulting in new stop codon; 1 event.

135	12 (.69%)	Mutation resulting in new stop codon; 4 separate events.

91	1 (.06%)	Mutation resulting in new stop codon.

^1^At least 2 nucleotides are changed when this stop codon is produced. Only 1 amino acid changes in the NS1 protein, at a relatively variable position. Phylogeny suggests approximately 13 different viruses arose in a variety of years with this stop codon after codon 197 in NEG8 ORF. ^2^142 codon ORF results from downstream AUG codon. Some of these ORFs would probably not be translated beyond a short peptide if the usual NEG8 initiating AUG is used.

The complete penetration of this mutation though the human influenza A viruses, which extends the NEG8 ORF to 216 codons, strongly suggests that it is providing a selective advantage to the virus. To exclude the possibility that this mutation was functioning through an effect on the NS1 protein, which is encoded by an overlapping (opposite direction) gene (Figure 1), we examined the sequence and variability of this site in the NS1 gene. This mutation changes the highly conserved NS1 codon 88 from CGC to CGA, but since both encode the amino acid arginine, this mutation apparently has no effect on the NS1 protein.

The next analysis examined the distribution of NEG8 ORF sizes, defined as the longest ORF on the genomic strand of segment 8, in non-human influenza A viruses post-1950. For avian influenza A viruses, 2646 (including avian-derived, but isolated from humans, H5N1) had a NEG8 ORF of <110 codons, 90 had a 110 <NEG8 ORF<155 codons, 16 had NEG8 ORF = 167 codons and 10 had NEG8 ORF>167 codons. For swine influenza A viruses, 64 had NEG8 <140 codons, 184 had NEG8 = 167 codons, 2 had NEG8 = 216 codons. For non-human/non-avian/non-swine influenza A viruses, all 221 had NEG8 ORFs that were <135 codons; a virus isolated from mink had a 167 codon NEG8 ORF (most probably a swine-derived virus) and a virus isolated from a giant anteater has a 216 codon NEG8 ORF (probably a human-derived virus). Thus it appears that the large, 216 codon ORF NEG8 is primarily associated with human flu A viruses, although a significant number of swine viruses possess the 167 codon NEG8 ORF similar to that which was circulating in the human population pre-1948. 9 avian viruses had a NEG8 ORF of 216 codons and 1 had a NEG8 ORF of 172 codons. These all had the same TAG > TAT change at the STOP codon after codon 167 as discovered in the human viruses and the serotypes were H9N2 (9) and H6N1 (1). The 2 swine viruses that contained a 216 codon NEG8 ORF also had the same change as the human viruses and were serotypes H1N1, isolated in 2007 (China, Accession No. FJ415613; A/swine/Zhejiang/1/2007(H1N1)), and H3N2, isolated in 2004 (Thailand, Accession No. AB434372; A/swine/Ratchaburi/NIAH59/2004(H3N2)).

We were also curious whether an ORF similar to NEG8 existed in any of the influenza B or C viruses. From the flu B and C viruses in the NCBI Influenza Virus Resource, the longest ORF on the negative strand of the NS coding segments were 103 and 109 codons respectively, and were found in the middle and 3' end of the negative strand, respectively. The predicted proteins from these ORFs had no significant similarity to the flu A NEG8 predicted protein.

### Bioinformatics evidence for a novel influenza A virus ORF

Considerable work has been performed to try and predict the significance of open reading frames and the likelihood of them being protein-coding; much of this has focused on the ratio of substitution rates at non-synonymous and synonymous sites [33]. Some work has also been applied to finding overlapping genes in viruses [34-36]. However, analysis of the influenza A virus segment 8 has several complicating factors, 1) the entire segment is only about 890 nucleotides, 2) NS1 and NEP genes overlap in two separate regions, 3) the NEG8 ORF overlaps both NS1 and NEP genes, and 4) the NEG8 ORF is on the opposite strand to the NS1 and NEP genes. Thus, these multiple overlaps of the 3 ORFs preclude the use of standard analyses. We therefore took the approach of directly examining nucleotide positions within segment 8, which had acquired mutations that were *fixed *in the human influenza A virus population during the last 60 years. Such mutations occurred at a single time point and did not very over subsequent years. For this period, human and avian virus sequences were available from 58 and 31 different years, respectively. More than twice as many mutations were fixed in the human segment 8 sequences than those derived from avian sources (a single avian segment 8 genotype (1E) was used as determined at the FluGenome genotyping resource; http://www.flugenome.org/). In the human viruses, these fixed single nucleotide substitutions resulted in 22 amino acid changes in the predicted NEG8 polypeptide sequence, and of course, there was no introduction of new stop codons because the 216 codon NEG8 ORF was maintained (Table 2). In contrast, in the avian viruses, the fixed single nucleotide substitutions resulted in 10 amino acid changes in the region equivalent to the NEG8 ORF, one of which was the introduction of a new stop codon in this reading frame (Table 3). This data, which shows that there are more mutations that both 1) change amino acid coding in NEG8 and 2) subsequently fixed or perpetuated in the virus population supports the hypothesis that the NEG8 ORF is conserved in human influenza A viruses and not in avian influenza A viruses. Furthermore, it suggests that the human influenza A virus NEG8 ORF is under positive selection.

**Table 2:** Nucleotide positions (aligned) of fixed mutations in human influenza A virus genomes

Position	NS1 protein	NEG8 reading frame	NS2 protein
183	N>D	F>S	

189	E>K	S>L	

202	R>H	R>W	

215	no change	no change	

218	no change	no change	

221	no change	P > L	

226	R>K	L>F	

245	no change	no change	

271	A>V	A>T	

326	no change	no change	

327	N>D	S>F	

341	no change	M>I	

361	A>E	A>S	

365	no change	no change	

374	no change	I>M	

383	I>M	no change	

392	no change	no change	

401	D>E	L>F	

404	no change	no change	

413	I>M	no change	

414	no change	S>N	

437	no change	no change	

449	no change	no change	

455	no change	no change	

456	L>I	R>M	

459	I>V	I>T	

506	no change	no change	

522	L>F	R>K	

538	N>I	L>I	

616	L>I	V>F	

636	no change	P > L	

657	no change	L>P	no change

668	S>L	no change	S>L

702	V>I	I>T	no change

703	I>A	T>A	no change

723	N>D	F>S	no change

Of 36 fixed single-nucleotide mutations in the avian influenza A viruses in the region of genome segment 8 spanning the region equivalent to the NEG8 ORF, 11 had no effect on NS1, NS2 or NEG8 reading frames, 17/36 had no effect on NS1, 4/5 had no effect on NS2. 22/36 mutations resulted in a change in the predicted amino acid sequence of the NEG8 protein

**Table 3:** Nucleotide positions (aligned) of fixed mutations in avian influenza A virus (segment 8 genotype 1E) genomes

Position	NS1 protein	NEG8 reading frame	NS2 protein
169	S>N	no change	

239	no change	no change	

311	no change	no change	

341	no change	I>M	

350	no change	no change	

379	R>K	no change	

404	no change	no change	

407	no change	I>M	

447	no change	S>N	

538	M>V	I>T	

618	no change	R>I	L>I

625	K>R	F>S	N>D

661	P > L	G>S	L>F

677	K>N	Y>*	S>I

729		I>T	no change

780		Q>R	no change

Of 16 fixed single-nucleotide mutations in the avian influenza A viruses in the region of genome segment 8 spanning the region equivalent to the NEG8 ORF, 5 had no effect on NS1, NS2 or NEG8 reading frames, 8/14 had no effect on NS1, 2/6 had no effect on NS2. 9/16 mutations resulted in a change in the predicted amino acid sequence of the NEG8 protein.

An alignment of the 38 avian influenza A virus segment 8 sequences reveals that approximately 52% of the nucleotides are conserved (100% identical) in every virus. The conservation among the 58 human sequences collected over the same time span is much greater, with 75% of the nucleotide positions perfectly conserved. This higher conservation may be the result of an additional selection pressure requiring the maintenance of a 3^rd ^gene, the 216 codon NEG8 ORF, in human viruses. This hypothesis is supported by the fact that the point mutations (not fixed in the population) that appeared in the 38 avian influenza A virus segment 8 sequences resulted in the generation of stop-codons within the NEG8 reading frame through at least 7 independent events (stop-codons at the same position in sequences from neighboring years were only counted once). A focused analysis of natural selection in human H3N2 influenza A viruses [36] similarly also revealed a reduction in the number of variable nucleotide positions in the region where the NS1 gene overlaps with the NEG8 ORF compared with the other non-overlapping gene regions. The number of variable nucleotide positions in the NS1-NEG8 overlap region was similar to that observed for the NS1-NEP overlap region [36]. As noted above, the overlapping ORFs in segment 8 make codon use analysis untenable, but we did observe that the ratio of NEG8 codons (1:28) that are scored as low "relative adaptiveness" for human codon use was no different to that found for the 2 genes on the opposite RNA strand (data not shown), this was approximately 1.6 times higher than human influenza A virus genes that do not overlap other ORFs.

### Evidence for the expression of FluA NEG8 ORF

With the above data, we hypothesized that certain influenza A viruses encode a novel protein, translated from a genomic sense copy of segment 8 and that this protein provides a selective advantage to the virus in a human host. Since the NEG8 ORF has been maintained in human influenza viruses since at least 1918, first as a 167 codon ORF and from 1948 as a 216 codon ORF, we predict that both forms of the protein product should be functional in humans. In addition to the absence of the NEG8 ORF (167 or 216 codon variants) from almost all non-human viruses, experiments using mouse-adapted human influenza A viruses also indicate that NEG8 may only function in humans. First, examination, by construction of specific genetic reassortments, of a mouse-adapted human flu A virus (A/FM/1/47-MA) indicated that segment 8 did not affect virulence in mice [37]. Second, introduction of the 1918 segment 8 into a mouse adapted virus resulted in attenuation of the virus in mice, rather than enhancing pathogenesis [18].

Although the maintenance of a 167 and later 216 codon ORF over almost 100 years in an RNA virus renowned for its variability (1.94+/-0.09 × 10^-3 ^substitutions/nucleotide site/yr [38]) is strongly indicative of an important function for the product of the NEG8 ORF, it doesn't prove that it is actually translated into protein in infected cells. Fortunately, there is biological evidence in the literature that this ORF is indeed translated into protein. In a study that mapped the CTL epitope repertoire of a 1934 (A/Puerto Rico/8/34) human influenza A virus, Zhong et. al. [39] first predicted (SYFPEITHI software, [40]) a murine H-2 D^b^/K^b ^CTL epitope (GGLPFSLL) within the 167 aa protein translated from the NEG8 ORF (denoted HP, hypothetical protein, in the paper) of this virus and then confirmed that CTLs isolated from mice infected with this virus responded by producing IFN-g when presented with the pure peptide [39]. In an IFN-g-ELISPOT assay, this NEG8 peptide ranked 5^th ^most effective IFN-g inducer of a group of 13 peptides that included a series of the most potent flu CTL epitopes. In another assay, which measured intracellular IFN-g, the NEG8 peptide induced a response in 1.5, 2.5 and 4.0% of CD8^+ ^T cells ranking 10^th^, 12^th ^and 4^th ^in a group of 16 peptides, which again included several known strong IFN-g inducers. In these two experiments, the authors recognized peptides as positive inducers of intracellular IFN-g if they produced at least 3-fold higher activity than background [39]. However, not only did the NEG8 peptide surpass this cut-off by a considerable margin, but it was also more potent than several proven immunogenic CD8^+ ^T cell epitopes. Our interpretation of this data is that during the influenza A infection, the NEG8 ORF was translated into a sufficient quantity of protein to induce a CTL response to this peptide. Several other NEG8 ORF peptides were also predicted to bind MHC by the SYFPEITHI program, (MHC Binding IDs 1006280, 1006282, 1006966, 1006969 and 1006970; Immune Epitope Database and Analysis Resource [41]) and shown to bind MHC molecules; however, discussion of these peptides was not included in the paper. Using BLASTP, we could not find any perfect matches to the 8 aa epitope sequence.

### Prediction of structure/function for influenza A virus NEG8 polypeptide

Although similarity searching using the more sensitive of the BLAST type programs [42] and the HHSearch tools [43,44], which searches for similar protein profile patterns, have often been very useful for generating novel hypotheses regarding protein function, these tools are in fact searching for distant similarities rooted in common ancestry. However, since the influenza A virus NEG8 ORF appears to have developed secondary to the NS1 and NEP genes on segment 8 of the viral genome, it is likely that there are no ancestral genes to be found. Therefore it was not surprising that our PSI-BLAST searches and analysis with HHSearch failed to find any distantly related protein sequences. We believe that the previously noted [30] similarity of the predicted NEG8 protein and a *Tetrahymena *protein (Accession no. Q950Z5) is spurious and the result of matching of the hydrophobic signal sequence of NEG8 protein and the highly skewed amino acid composition of the *Tetrahymena *protein.

Subsequent bioinformatics analyses focused on searching for functional polypeptide motifs. For this series of experiments the 167 codon NEG8 ORF of the 1918 strain of influenza A virus and the 216 codon NEG8 ORF of 2 human H1N1 viruses (1950, Access. No. K00576; 2006, Access. No. CY017375) and 2 human H3N2 viruses (1970, Access. No. AY210306; 2006, Access. No. CY016999) were used. InterProScan [45], flagged only potential signal sequences and transmembrane domains (TMs) in these 5 proteins. ScanProsite [46,47], found no hits when searching for non-frequent motifs, and none of the sites of common patterns (e.g. N-linked glycosylation site) were absolutely conserved among the 5 proteins.

To evaluate the significance of the predictions of a signal peptide and TMs we retested the 5 proteins with multiple software tools including SignalP v3.0 (SignalP-NN and SignalP-HMM) [48,49], Phobius [50], SPOCTOPUS [51], TOPCONS [52], TMHMM [53] and SIGNAL-BLAST [54]. Although there were some minor discrepancies between the results of these programs, Figure 3 shows the consensus organization with the presence of a signal peptide and 2 TMs. The variations (some proteins, some tools) were 1) inability to distinguish the signal peptide from the first TM and 2) the occasional prediction of a third TM at the end of the 216 aa protein. The 5 proteins range from approximately 78-92% pair-wise aa identity and the consistent prediction of the signal peptide and TMs suggests that this organization should be considered as a potential structural model. It is interesting to note that the 49 codon extension to the 167 codon ORF following the mutation in approximately 1947 would result in the simple extension of the C-terminal "Outside" domain of the predicted NEG8 proteins (Figure 3).

## Discussion

Our hypothesis proposing that a novel gene is encoded by the genomic sense strand of the human influenza A virus segment 8 RNA has a number of significant implications. The first and perhaps simplest consequence is that this genome segment would be ambi-sense, a unique feature in the Orthomyxoviruses. Second, if the maintenance of the 216 codon NEG8 ORF in essentially all human influenza A viruses is because it is translated into a polypeptide that confers a selectable advantage upon the virus, then the conclusions derived from many of the published experiments that used deletion and site-specific mutations to investigate the role of the NS1 and NEP proteins on the replication and virulence of human influenza A viruses would need to be re-evaluated because many of these engineered mutations also interfere with the integrity of the NEG8 ORF [55-59]. The third important implication relates to the fact that this NEG8 ORF is almost universally linked to human influenza A viruses and the associated consequences of its introduction into an avian or swine influenza A virus through co-infection and re-assortment. The 1957 (H2N2) and 1968 (H3N2) human influenza A pandemics arose from antigenic shift events following the introduction of NA and/or HA gene segments into the human influenza A virus circulating at the time [1,32,60] with no exchange of genome segment 8; the same segment 8 genotype has circulated in the human population since, at least, the 1918 pandemic and is therefore presumably well-adapted to provide viral fitness when the virus is replicating in humans. Currently, there are 2 zoonotic influenza A viruses, avian-derived H5N1 and swine-derived H1N1, that are potential *pandemic viruses *and one must consider the effect of the introduction of a human segment 8 into one of these viruses. The avian-derived H5N1 virus is highly pathogenic but transmits to and among humans poorly [14], where as the swine-origin H1N1 virus appears to be far less pathogenic but transmits easily among humans [61]. Since the 167 and 216 codon ORFs are absent from both the H5N1 avian influenza viruses and the new swine-derived H1N1 viruses, the introduction of this NEG8 ORF into either of these viruses by reassortment with a human influenza A virus or by mutation could have very dire consequences. If the highly pathogenic H5N1 avian virus acquired a human influenza A virus genome segment 8 with the 216 codon NEG8 ORF, it might become more easily transmitted among humans; alternatively, if the swine-derived H1N1 virus acquires human influenza A virus genome segment 8 from a currently circulating human H1N1 or H3N2 virus then the novel virus might have increased virulence associated with the NS1 virulence factor [12,62] or the 216 codon NEG8 ORF. Both of these scenarios potentially have enormous consequences for human health, in part because of the lack of previous exposure of humans to these strains by natural infection or vaccination.

**Figure 4:**
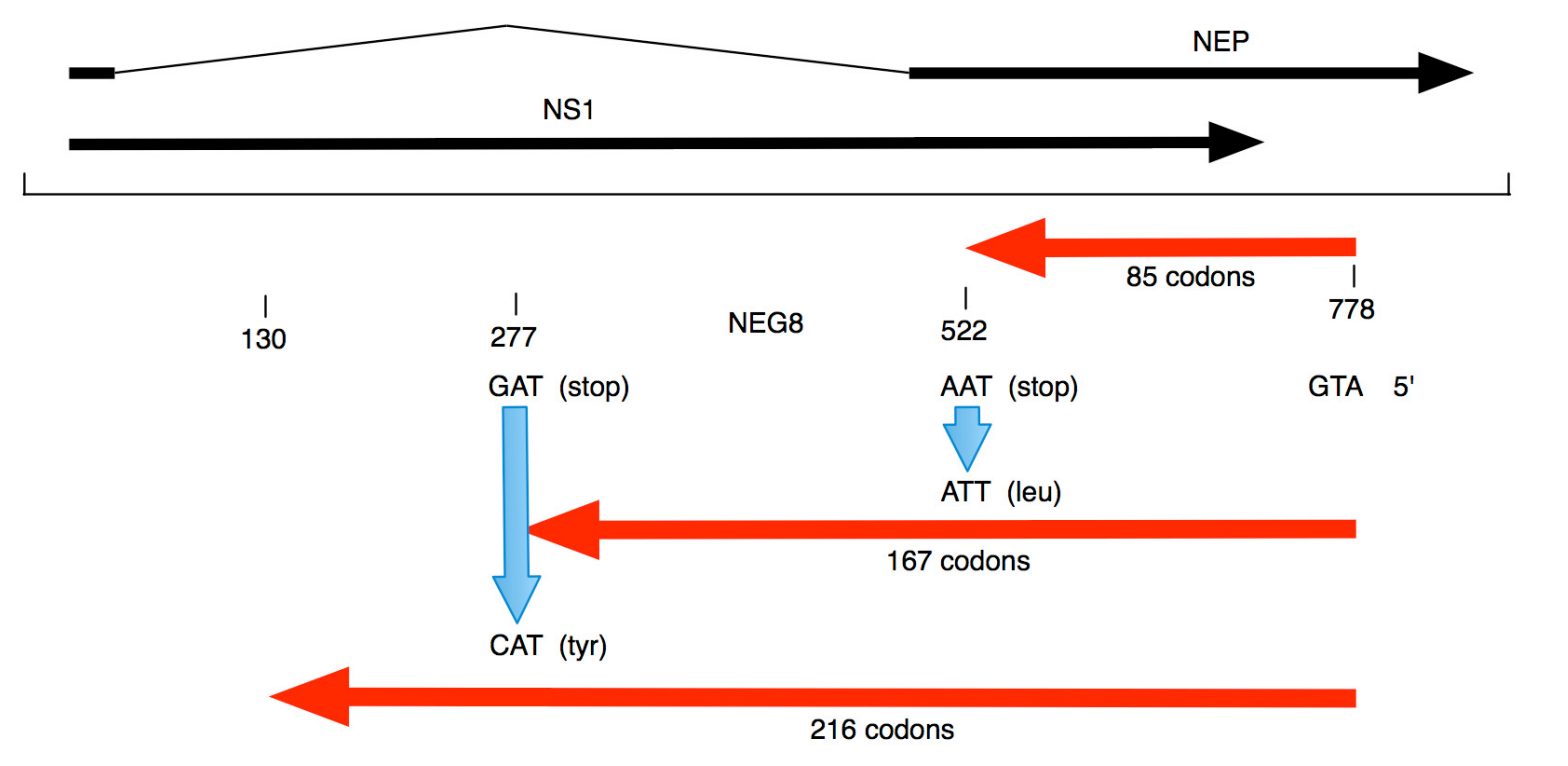
**Organization of negative strand ORFs in a 2009 swine-derived H1N1 influenza A virus**. The NEG8-like open reading frames are shown as red arrows. Blue arrows indicate positions where single nucleotide changes are required to extend the 85 and 167 codon NEG8 ORFs. NS1 and NEP genes are shown in black.

However, the latter appears more likely because both of these H1N1 virus types are apparently now replicating efficiently in humans. Analysis of the sequence of the swine-derived H1N1 genome segment 8 revealed that it contains the same initiating ATG as the human NEG8 ORF and only requires the removal of 2 stop codons, each by a single nucleotide change, to generate the 216 codon NEG8 ORF (Figure 4). Only 1 nucleotide change is required to produce the 167 codon variant of the NEG8 ORF. The product of a swine-derived H1N1 NEG8 ORF constructed in this way would share 71% amino acid identity with the current human NEG8 protein over the 216 aa.

The rather sudden and total replacement of the 167 codon NEG8 ORF by the 216 codon NEG8 ORF after 1947 is especially intriguing. It is interesting to note that a human influenza A virus H1N1 *pseudopandemic *(low death rates) also occurred in 1947 [63]. This has been attributed to a significant, but non-shift, antigenic change in HA and NA proteins [64]. However, due to a lack of genomic sequence information, it is impossible to determine whether the coincident change to the 216 codon ORF was involved in creating a virus capable of spreading world-wide or whether the pseudopandemic merely coincided with the genetic change and had the effect of seeding that virus type throughout the world. However, the maintenance of the 216 codon NEG8 ORF over many years appears to be a very different matter; very few human non-216 codon ORFs have been isolated and none have persisted, whereas there are multiple examples of the appearance of stop codons in this reading frame for the avian viruses. It is also notable that human-specific selection on amino acid sequence has been observed in the influenza A virus M protein [65].

Since there is no recognized mechanism for the translation of ORFs encoded on the genomic strands of influenza viruses, an obvious question is "how could a NEG8 protein be produced?". The answer is that there are already a number of examples in the literature describing the detection of CTL epitopes from *non-traditionally *derived proteins (reviewed in [66]), which are produced at low levels. Interestingly, in addition to PB1-F2, the production an additional influenza A peptide (N40, a fragmented version of PB1 protein) has been recently demonstrated[67]. Mechanisms for generation of rare proteins include ribosomal frameshifting (e.g. from the influenza NP gene [68]), non-AUG initiation of translation (e.g. from the influenza HA gene [69]), initiation codon scanthrough (e.g. from the influenza NP gene [70]) and internal initiation of translation (e.g. Hepatitis C virus F protein [71]). Clearly, some of these mechanisms (those that utilize a normal viral mRNA) are not appropriate for translation of an ORF from an influenza A virus genomic RNA, however, these data reinforce the fact that molecular processes are not perfect and that *errors *in transcriptional and translational events are likely to lead to the production of small amounts of such *non-traditional *polypeptides, which in turn provide targets for evolutionary forces and may lead to the eventual evolution of novel genes such as PB1-F2 [36] and NEG8. Another mechanism, present in some viruses, is the use of Internal Ribosome Entry Sites (IRES), which are complex structural features present in mRNAs [72,73] that provide a mechanism for initiation of translation independent of a 5'-CAP; although no such structure is obvious in the 5' end of the genomic RNA of segment 8, the presence of IRES elements are very difficult to predict computationally [74] since they are extremely variable in sequence [75-78].

Normal influenza A virus mRNAs, but not genomic RNAs, are poly-adenylated by stuttering of the polymerase, which is a process integrated with of mRNA transcription[79]. Although polyadenylation stimulates mRNA translation, it is not absolutely required[80]. Therefore the high levels of influenza A virus genomic RNA in infected cells, could be sufficient to allow some translation of the NEG8 ORF even if the RNA is not poly-adenylated.

Finally, although this 216 codon NEG8 ORF is very tightly associated with human influenza A virus infections and may have been a factor in the 1947 pseudopandemic, its role in viral pathogenesis may be very difficult to unravel. First, the NEG8 ORF overlaps with NS1 and NEP genes on segment 8, which makes it a difficult target for deletion and mutagenesis studies, and second, because the NEG8 ORF is not absolutely essential for replication of human influenza A virus in either its 167 or 216 codon form (PB1-F2 and N40 are also not essential) nor present in most animal and avian influenza A viruses, it may be very difficult to correlate an observable phenotype with its presence using animal models.

## Conclusion

There is an unusually long (648 nt) ORF on the genomic (negative) strand of segment 8 of current human influenza A viruses. The very high degree of conservation of this ORF and the detection of a CTL response to a peptide fragment of the predicted protein suggests the ORF is expressed. The predominant association of this ORF with human influenza A viruses indicates that an expressed protein may only be an advantage to influenza viruses replicating in humans; this could have very significant implications if the swine H1N1 influenza A virus, which is currently causing a human pandemic mutated to acquire this novel ORF.

## Methods

### Sources of influenza A virus sequences

Human influenza A virus genome sequences were selected and collected from the Influenza Virus Resource at NCBI [81]. Avian influenza A viruses of a single segment 8 genotype were selected using FluGenome, a web tool for genotyping influenza virus [82]. Segment 8 sequences from all genomes were used (>2000), with the exception of 1) duplicates, 2) those with severe truncations and 3) those with frameshift errors (in NS1 or NS2 genes) that were assumed to be sequencing mistakes. Duplicate and truncated sequences (<5%) were avoided using NCBI selection parameters. A local script was used for translating the longest ORF on the genome strand.

### Bioinformatics software

MUSCLE was used for generating multiple sequence alignments [83], which were viewed and edited using the Java program Base-By-Base [84] via the Viral Bioinformatics Resource Center [85]. The ORF Finder software at the National Center for Biotechnology Information (USA) was used to visualize the length of the NEG8 ORF.

SignalP v3.0 (SignalP-NN and SignalP-HMM) [48,49], Phobius [50], SPOCTOPUS [51], TOPCONS [52], TMHMM [53] and SIGNAL-BLAST [54] were used to predict signal peptide and transmembrane domains in NEG8.

## Competing interests

The authors declare that they have no competing interests.

## Authors' contributions

CU conceived the idea for the work, performed some analyses and wrote the manuscript. MC and JT performed data collection and analyses.
